# A Rapid and Simplified Approach to Correct Atmospheric Absorptions in Infrared Spectra

**DOI:** 10.1021/acs.analchem.4c03594

**Published:** 2024-10-31

**Authors:** Waseem Ahmed, Eleanor L. Osborne, Aneesh Vincent Veluthandath, Ganapathy Senthil Murugan

**Affiliations:** Optoelectronics Research Centre, University of Southampton, Southampton SO17 1BJ, U.K.

## Abstract

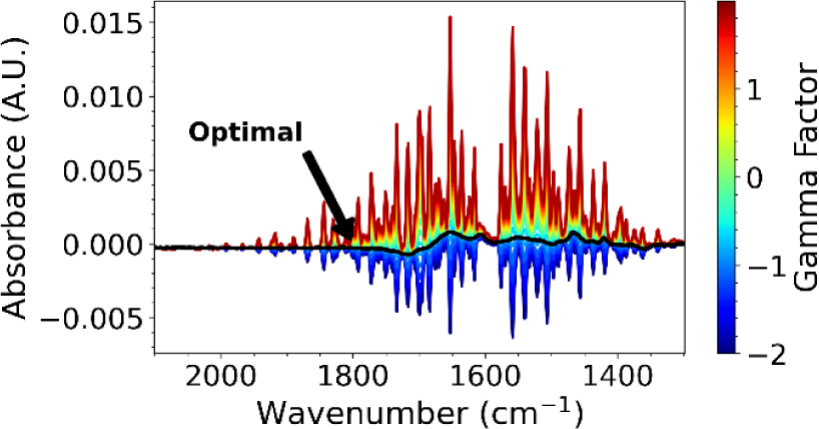

Infrared (IR) spectroscopy is a powerful analytical technique used to identify and quantify different components within a sample. However, spectral interference from fluctuating concentrations of water vapor and CO_2_ in the measurement chamber can significantly impede the extraction of quantitative information. These temporal fluctuations cause absorption variations that interfere with the sample’s spectrum, making accurate analysis challenging. While several techniques to overcome this problem exist in the literature, many are time-consuming or ineffective. We present a simple method utilizing just two sample spectra taken sequentially. The difference of these spectra, multiplied by a scaling factor, determined by minimization of the point-to-point spectral length, provides a correction spectrum. Subtracting this from the spectrum to be corrected results in a fully corrected spectrum. We demonstrate the effectiveness of this method via the improved ability to determine analyte concentration from corrected spectra over uncorrected spectra using a partial least square regression (PLSR) model. This technique therefore offers rapid, effective, and automated spectral correction, which is ideal for a nonexpert user in a clinical or industrial setting.

## Introduction

Fourier transform infrared (FTIR) spectroscopy has become an indispensable analytical technique for researchers. Molecules, including important biomarkers and atmospheric gases, have a characteristic set of absorptions in the mid-infrared depending on their bond vibrations. This enables a “fingerprint” spectrum to be collected, providing an insight into the molecular composition of a sample. Consequently, FTIR spectroscopy is an ideal candidate for implementation in point-of-use sensing, including in environmental and medical applications.

However, the measurement environment can negatively impact spectral quality, which poses a particular issue when measuring molecular vibrations of proteins, lipids, and carbohydrates. Both water and CO_2_ absorb strongly in the mid-infrared (see Table 1). For liquid phase measurements, attenuated total reflection (ATR) may be used to reduce the path length of light in water by probing the solution with only an evanescent field. Yet, even in a vapor form, water presents a problem for both transmission and ATR measurements. Usually, a background spectrum is taken prior to sample measurement, enabling the effects of any background absorptions to be removed from the sample spectrum. Nonetheless, changes in water vapor and CO_2_ concentrations within the measurement chamber result in absorbance changes causing features of their spectra to reappear in the sample spectrum.^1^ These residual absorption features can obscure the sample spectrum, making the extraction of quantitative information relating to the sample challenging. Purging using nitrogen is one solution used in a laboratory setting to overcome variations in atmospheric conditions. Despite that, the necessity to open the chamber following a background scan to insert a sample results in alteration of the chamber’s CO_2_ and water vapor content. Subsequent purging may take a considerable time to restore atmospheric conditions at which the background scan was taken or may not be able to do so at all. Furthermore, nitrogen purging may not always be an option, for example, when using compact spectrometers in “remote” locations away from a laboratory, making a method of correction for environmental absorptions immensely important. In the following sections, discussion of water vapor interference should be assumed to apply to interference from any other vapor or gas whose concentration varies temporally in the measurement chamber including carbon dioxide.

**Table 1:** Infrared Spectral Regions in Which Water Vapor and CO_2_ Absorb^1^

Molecule	Wavenumber region (cm*^–^*^1^)	Vibration assignment
CO_2_	600 to 914	Bending
CO_2_	2208 to 2442	Asymmetric stretch
CO_2_	3701 to 3731	Combination of bend and stretch vibrations
CO_2_	3602 to 3627	Combination of overtone and stretch vibrations
H_2_O	1205 to 2072	Bending
H_2_O	3231 to 4000	Symmetric and asymmetric stretching

Two approaches have been taken previously to correct environmental interference in FTIR spectra. The first is to manually exchange humid/dry air in the measurement chamber to match the original water vapor concentration measured at the background scan, as performed by Chen et al.^2^ Similarly, Zhang et al. controlled laboratory temperature and humidity to minimize the effect of water vapor absorption on ATR–FTIR spectra of pericardial fluids.^3^ However, the time devoted to manually altering the conditions in the chamber is extensive. Therefore, obtaining spectra completely free from any environmental effects using such techniques is usually unrealistic and impractical, especially when considering a nonexpert user in a point-of-care setting measuring low concentration analytes.

Therefore, a second “software-based approach” provides advantages. A correction is applied to the spectra to remove the environmental effects while preserving the analyte spectrum. These correction methods have also been the subject of patents which correct the spectra from water and carbon dioxide effects at the point of collection.^4^ Perhaps the simplest software-based method to remove unwanted absorptions is a smoothing algorithm, such as a Savitsky–Golay algorithm^5^ or low-pass filter.^1^ Still, such a method is inherently flawed, as spectral information from the sample itself can be lost, particularly for weak absorptions in low concentration samples. Bruun et al. allude to the fact that biological sample spectra may include sharp features such as the amide I sub-bands of proteins that have similar line widths to water vapor;^1^ therefore, applying a low pass filter to remove the effects of water vapor will also distort required analyte spectral information.

The earliest effective methods for removal of water vapor absorption involved spectral subtraction, whereby a water vapor spectrum is multiplied by some constant and subtracted from the sample spectrum. However, manual selection of a constant to multiply with the water vapor spectrum is time-consuming,^6^ and variations in environmental conditions such as temperature can cause spectra to contain contributions from different rotational fine structure transitions. Algorithms to perform such calculations have been developed by using a variety of techniques. Bruun et al.^1^ and Bruździak^6^ utilized least-squares fitting to calculate multiplication coefficients for several calibration vapor spectra added together to remove vapor effects from spectra. This helps overcome the issue of temperature dependent absorptions; however, both techniques required measurement of multiple calibration spectra of environmental gas/vapor absorptions, which is time-consuming. Reid et al.^7^ and Erik and Jean-Marie^8^ both utilized Fourier deconvolution to enable removal of water vapor absorptions from protein spectra. Perez-Guaita et al.^9^ measured a reference spectrum for each interferent gas/vapor prior to recording analyte spectra. Relative absorbance of the water vapor at specific prespecified wavenumbers was then calculated before subtraction of a scaled water vapor spectrum to correct the analyte spectrum. Alternatively, Kojić et al.^10^ used models of ambient air absorption to remove these features from modeled spectra.

## Atmospheric Correction Method

We propose a method to correct for spectral interference by subtracting scaled spectra containing temporally changing atmospheric gas/vapor absorptions based on minimization of point-to-point spectral length. The point-to-point length of the spectrum is the distance between consecutive data points summed over the full spectrum. For the background corrected blank spectrum, the point-to-point length of the spectrum is the minimum possible length of a spectrum and has the form of a straight line. If an analyte is placed in the beam path, this will result in increased absorbance for regions of the spectra associated with the vibrational modes of the analyte. Consequently, the point-to-point length of the spectrum will increase from that of the blank spectrum case. In a similar manner, a change in the chamber conditions of water vapor concentrations results in an increase in the point-to-point spectral length. Reducing the point-to-point spectral length associated with the absorbance profile of a particular interferent in the spectrum has the effect of removing it from the spectrum. This method greatly reduces the measurement overhead for spectral correction compared to similar literature techniques and can be implemented as part of spectral postprocessing.

The key steps required to perform the correction are:

1 Measure a background spectrum. This may be air only, or the solvent used to dissolve the analyte.
2 An analyte spectrum to be corrected is recorded at *t*_1_ and designated Spectrum 1 (*S*(*t*_1_)), and an analyte spectrum recorded in immediate succession (at *t*_2_) is designated Spectrum 2 (*S*(*t*_2_)).
3 Spectrum 2 (*S*(t_2_)) is subtracted from the spectrum to be corrected (*S*(*t*_1_)) to obtain a difference spectrum (*D*) in spectral regions affected by atmospheric gas/vapor absorptions (shown in Table 1).
4 The point-to-point spectral length is minimized by multiplying the difference spectrum by a scaling factor and subtracting it from the spectrum over the region affected by the water vapor. The scaled difference spectrum should reflect the true environmental conditions present when the background spectrum was measured. This produces a corrected spectral region called the minimized length spectrum (*S*_m_).
5 *S*_m_ overwrites the corresponding region in the original spectrum to produce a fully corrected spectrum (*S*_a_).

**Figure 1:**
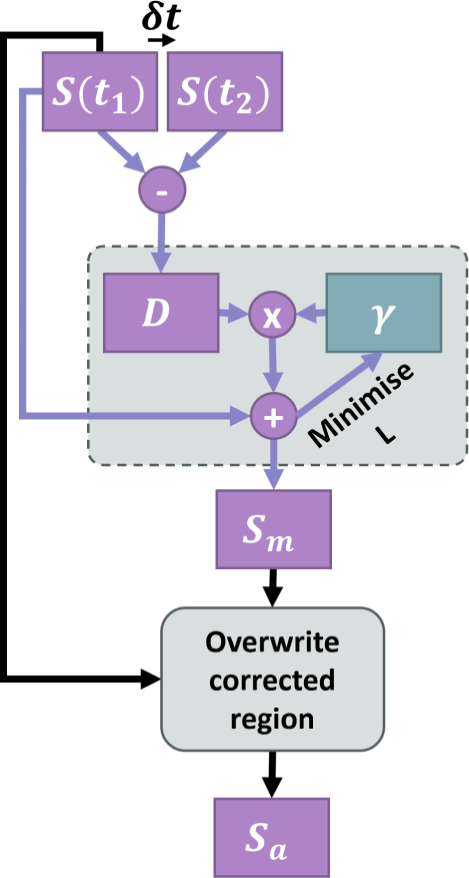
Flowchart showing the key steps of the correction algorithm. Spectrum 2 (S(t_2_)) is the repeated analyte spectrum taken immediately before or after Spectrum 1 S(t_1_), D is the difference spectrum produced by subtracting S(t_2_) from S(t_1_), γ is a scaling factor applied to D, S*_m_* is the minimized point-to-point length spectrum and S*_a_* is the final corrected spectrum.

The flowchart in Figure 1 details these steps pictorially.

In an ideal case, Step 1 may be omitted. However, normally, if a background spectrum is required, it should be taken immediately prior to the analyte spectra. If this is not the case, increasing instrumental drift, particularly if caused by a reference laser frequency shift, will result in the appearance of water vapor features in the recorded absorbance spectrum that do not change with time. This then becomes similar to the analyte spectra and will not be corrected. By ensuring that a new background is taken each time, this problem is avoided. To correct for environmental effects in a spectrum, we must isolate them from the analyte signal. We can consider the measured spectrum to be a sum of the analyte spectrum (*S*_a_) and spectra of interferent gases or vapors, including water  and carbon dioxide , multiplied by a coefficient (, , etc.) representing their time dependent concentration eq 1:


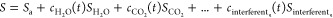
1

Given that the variation of concentration of each interferent with time cannot be assumed to be the same for all interferents, a spectral region corresponding to absorption by a single interferent is selected to reduce the number of unknown values in the equation (such spectral regions are shown for water vapor and CO_2_ in Table 1). Taking a region containing absorption by water vapor as an example, we are left with eqs 2 and 3 representing Spectrum 1 and Spectrum 2 taken sequentially
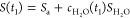
2
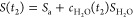
3

It is important to note that the spectrum of the interferent (in this case, ) is assumed to be time independent within the small time frame of the measurement of sequential spectra. Instead, the coefficient  is time dependent, representing a time dependent concentration of the interferent in the beam path. This is because we do not expect a significant change in ambient temperature to occur within this time frame, which would change the relative intensity of the water fine structure in  and invalidate the method. Therefore, the presented method would not be valid in situations with rapid temperature or pressure changes between measurements of spectra.

As *t* is different for each spectrum recorded, a difference spectrum (*D*) can be extracted by subtracting successive spectra in a region corresponding to water vapor absorption
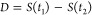
4where it should also be true that
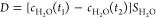
5

Rearranging eq 2
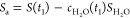
6where S_a_ can also be expressed as the sum of measured spectrum *S*(*t*_1_) and the difference spectrum (expressed in eqs 4 and 5) multiplied by some scaling factor γ, which is specific to the interferent being corrected for
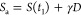
7

By equating eqs 6 and 7, eq 8 can be derived as
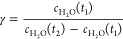
8

However, without previous knowledge of the value of , γ must be calculated numerically. To do this, each data point of the spectrum is mapped to Euclidean space, such that the spectrum is reconstructed in Euclidean space. Considering the spectrum as a series of data points connected by straight lines, the point-to-point length of spectrum (*L*) can be calculated using Pythagoras' theorem
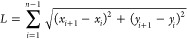
9where *i* is the index of the data point in the spectrum containing *n* data points. Each data point can be considered to have coordinates *x* and *y* in Euclidean space, which are unitless. A diagram demonstrating the calculation of eq 9 is shown in Figure S1. When all the contributions to the spectrum by water vapor are removed, it will have a minimal point-to-point spectral length. This can be established by measuring the point-to-point spectral length of the output spectrum post subtraction of the scaled difference spectrum (γ*D*), as per eq 7, and selecting the optimum γ that gives a minimum point-to-point spectral length.

Finally, the corrected region of the spectrum (*S*_a_) is rejoined to the original spectrum (*S*(*t*_1_)) and overwrites the original environmental absorbances. In order to do this, the absorbances at each end of the spectral region were matched. This process is repeated for each individual interferent, giving an independent value of γ for each. The advantage of this method is that multiple sample spectra are usually measured as the usual experimental protocol, so no additional work is required by the user to take calibration spectra.

## Methodology

### Collection of Spectra

An Agilent Cary 670 FTIR spectrometer with a DLTGS detector was used to record spectra with a resolution of 4 cm*^–^*^1^ (unless otherwise stated), with the sample chamber purged with N_2_ gas. A ZnSe ATR crystal was used for spectral acquisition of samples included in this paper: benzaldehyde, sodium acetate dissolved in water, and varying concentrations of sphingomyelin (SM) and dipalmitoylphosphatidylcholine (DPPC) in dichloromethane (DCM). A background spectrum was collected, followed by at least 8 sequential measurements of the sample. Atmospheric absorptions in spectra were then corrected utilizing the methodology set out above. For the purpose of validation of the presented spectral correction method, a number of ideal spectra were also collected. This was done by careful control of conditions in the chamber, such that the atmospheric conditions when the spectrum was measured were nearly identical to those at which the background spectrum was taken, as described by Chen et al.^2^ This means that these ideal spectra have negligible absorptions by water vapor or CO_2_, enabling them to be used as a comparison to the corrected spectra to validate our method.

## Results and Discussion

**Figure 2:**
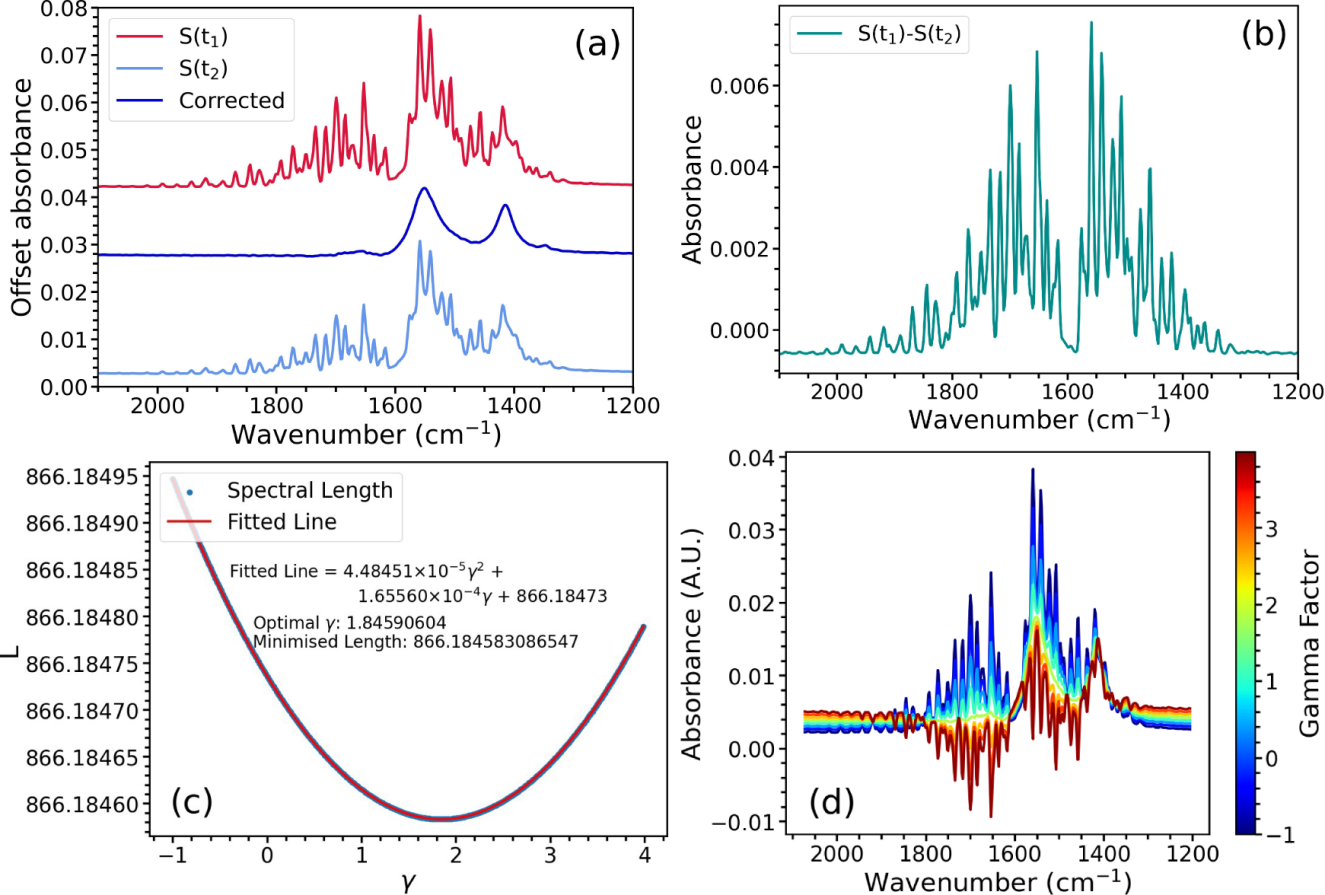
(a) Spectra 1 and 2 and corrected spectrum of 250 mM sodium acetate in water. Spectra 1 and 2 were collected using N_2_ purging but show clear interference from water vapor. The uncorrected spectrum is successfully corrected for water vapor absorptions. The spectra are offset for clarity. (b) Subtraction of Spectrum 2 from Spectrum 1 shown in (a) provides this difference spectrum. (c) The point-to-point length of the spectrum in the correction region plotted as a function of the value of γ showing the optimum value of γ at which the point-to-point spectral length is minimized. The equation for the fitted line is truncated to 5 decimal places. (d) The spectrum in the correction region plotted with different values of γ, demonstrating the effect of changing γ on the appearance of water vapor absorbance peaks.

Figure 2a shows spectra of 250 mM sodium acetate in water taken sequentially (Spectrum 1 and Spectrum 2) and background corrected to air. It can clearly be seen that the absorption by water vapor varies significantly between the spectra due to variations in the amount of water vapor in the beam path at each measurement. Subtracting the second spectrum from the first spectrum produces the difference spectrum shown in Figure 2b. Utilizing our methodology, the value of γ was then optimized to correct the uncorrected spectrum and produce the corrected spectrum shown in Figure 2a. The corrected spectrum is visibly smoother than the uncorrected spectrum (*S*(*t*_1_)), with the sharp absorptions due to water vapor removed to reveal features of the sodium acetate spectrum that could not previously be resolved.

The effect of different γ values on the point-to-point spectral length is illustrated in Figure 2c and the corresponding corrected spectra are shown in Figure 2d. The value of γ scales the difference spectrum, which is then subtracted from the uncorrected spectrum. Figure 2c shows that there is an optimal scaling γ factor, which results in a minimum length for the remaining spectrum. This can be solved analytically in Figure 2c to give the optimal γ value for the minimal spectral length (in the correction region).

### Edge Cases

While the method presented works effectively for the majority of spectra tested, we determined a number of “edge cases” that tested the efficacy of the techniques. These occur when an analyte, such as benzaldehyde, has particularly intense absorption peaks in a spectral region containing interference from water vapor. When this region is analyzed to find an optimum value for scaling factor γ, sharp intense peaks from the analyte result in the calculation being erroneous.

**Figure 3:**
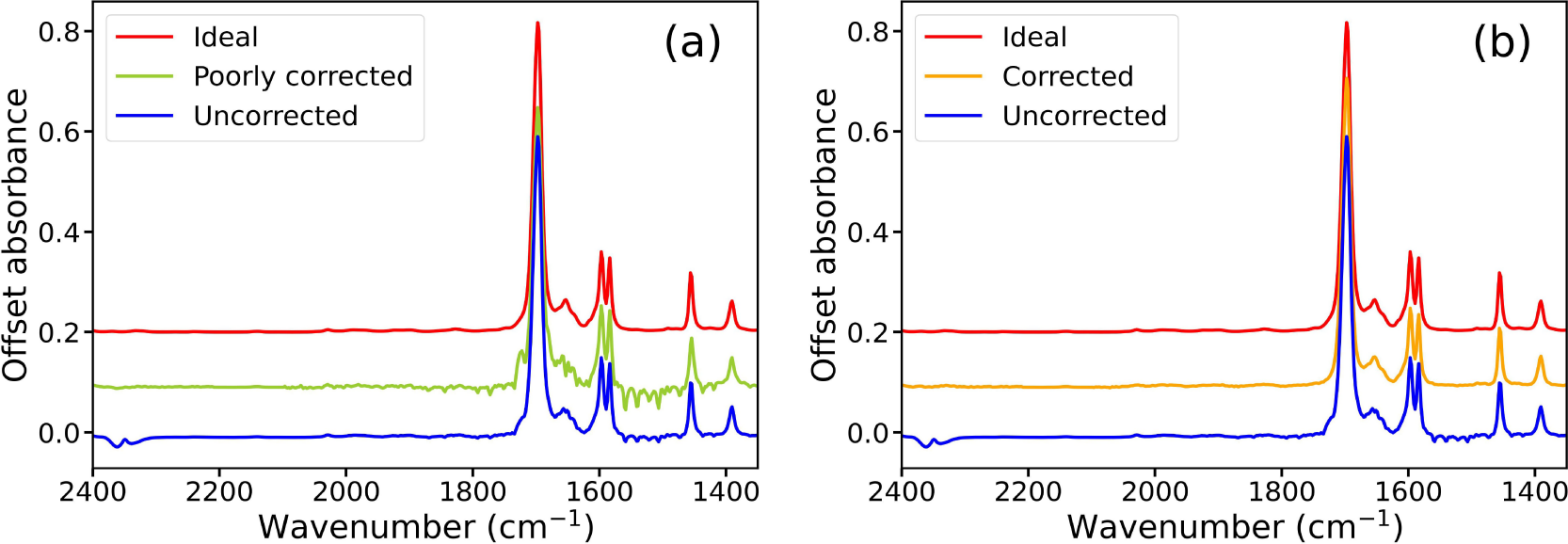
(a) Example of a benzaldehyde infrared spectrum background corrected to air that has been poorly corrected for water vapor absorption due to erroneous determination of γ. (b) Improved correction of the spectrum obtained by defining an alternative region that contains no sharp intense peaks over which γ was optimized. In each case, an ideal spectrum is shown for comparison.

To overcome these errors, the scaling factor can instead be determined by analyzing alternative spectral regions where the same atmospheric contaminant absorbs but which are free from intense analyte absorptions. For example, the 3231–4000 cm*^–^*^1^ region can be used as an alternative for the 1205–2072 cm*^–^*^1^ region for water vapor correction. As shown in Figure 3, correction of water vapor in the 1205–2072 cm*^–^*^1^ region is greatly improved in graph b compared to graph a by using a value γ derived from the 3231–4000 cm*^–^*^1^ region.

The sharp intense absorptions displayed by benzaldehyde, especially at approximately 1700 cm*^–^*^1^, result in erroneous calculation of γ in Figure 3a. This shows that a scaling factor found in one region of absorption by a particular gas/vapor can be implemented in another region for that same gas/vapor. This applies even in cases where the correction that is applied is subtle (see Supporting Information and Figure S2 for further details).

### Comparison of Uncorrected and Corrected Spectra

In order to determine the validity and effectiveness of this correction algorithm, a statistical comparison of the corrected and uncorrected spectra is required. The simplest method to do this is to determine the Pearson correlation coefficient at each wavenumber between corrected, ideal, and uncorrected spectra in regions of atmospheric correction. However, the spectra of the same analyte tend to be highly correlated even when atmospheric effects are present. To differentiate between them, Perez-Guaita et al.^10^ used the Fisher *Z* transform which normalizes the Pearson correlation coefficient, making it easier to differentiate between correlations that are all high. This method was used to test our algorithm initially, but spectral regions with no features (from the analyte or atmospheric absorptions) were found to be significantly different using the Fisher *Z* transform, which is clearly incorrect. The cause of this was pinpointed to instrumental drift. For the Fisher *Z* transform to be valid, the data at each wavenumber must be independently and identically distributed around some mean absorbance; spectral drift invalidates this.

**Figure 4:**
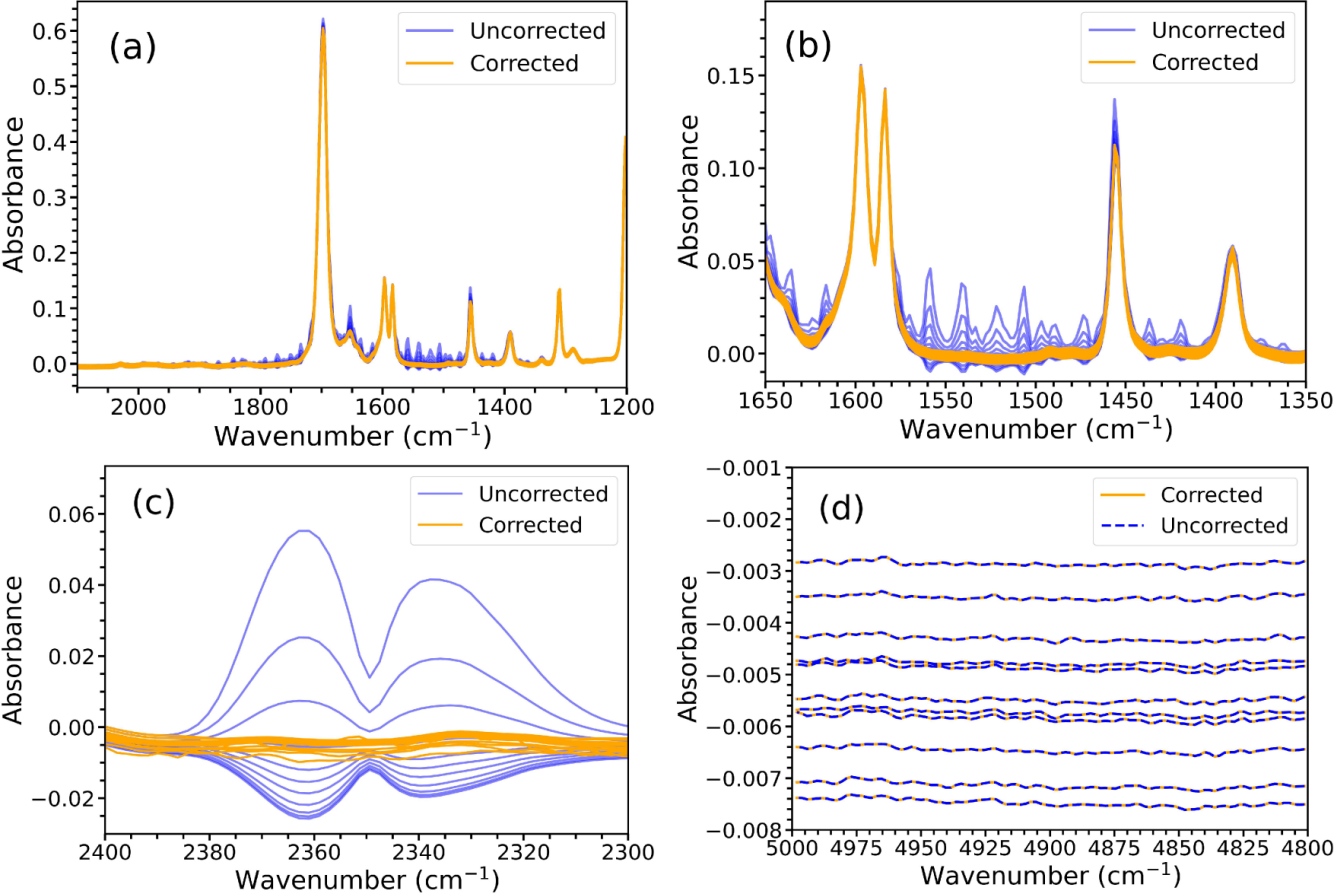
Regions of sequentially collected benzaldehyde spectra used to calculate the RSD metric to determine the efficacy of the correction method. (a) is a region that includes water vapor absorptions corrected in the 1205 to 2072 cm*^–^*^1^ region. (b) is a zoomed in view of (a). (c) is a region that contains CO_2_ absorptions corrected between 2208 and 2442 cm*^–^*^1^, and (d) shows a region with no analyte or environmental absorptions used to calculate a baseline standard deviation.

Therefore, a simple measure of relative standard deviation (RSD) is used to analyze the effectiveness of the correction algorithm. A large number of benzaldehyde spectra were collected using ATR–FTIR spectroscopy sequentially. The uncorrected spectra were corrected using the algorithm to produce the data shown in Figure 4. Three spectral regions were then chosen for comparison. To assess water vapor correction, the 1200 to 2100 cm*^–^*^1^ region (Figure 4a,b) was used, and the 2300 to 2400 cm*^–^*^1^ region was used for CO_2_ absorptions (Figure 4c). A region containing no spectral features from either atmospheric or analyte absorptions between 4800 cm*^–^*^1^ and 5000 cm*^–^*^1^ was chosen to provide a baseline standard deviation at each wavenumber for noise and experimental drift (Figure 4d).

**Figure 5:**
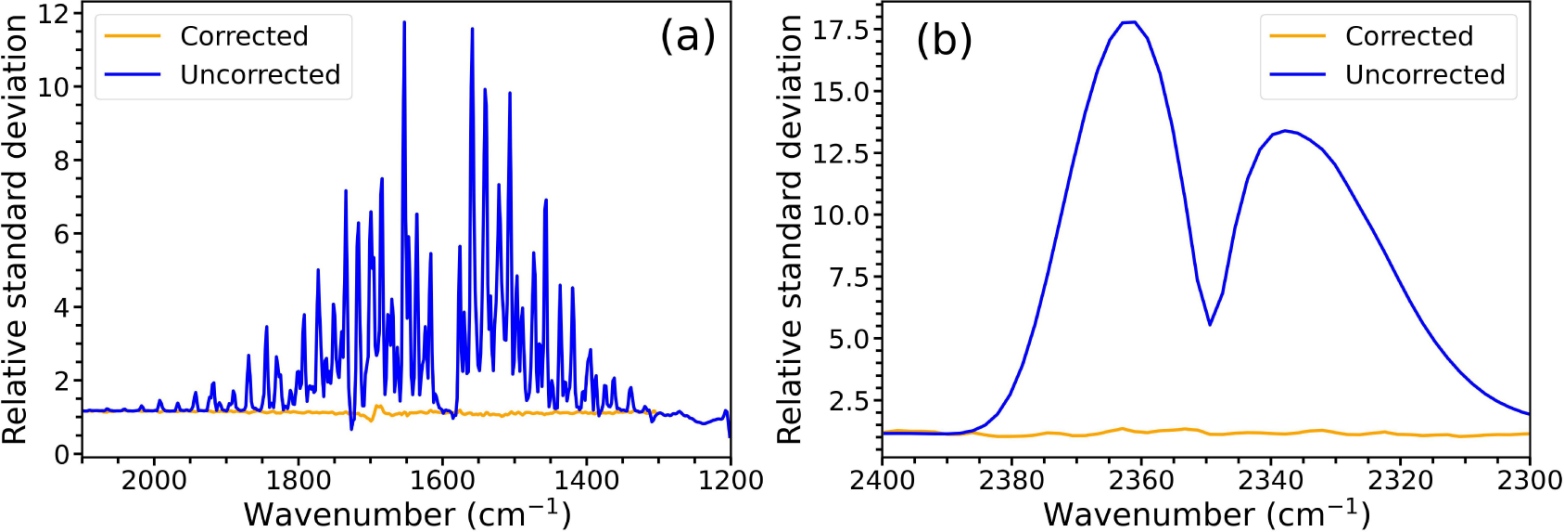
RSD calculated in (a) a water vapor absorption region, and (b) a CO_2_ absorption region for each set of corrected and uncorrected spectra of benzaldehyde. The corresponding spectra are shown in Figure 4.

The RSD was determined by dividing the standard deviation at a point in a region of water vapor or CO_2_ absorption by the mean standard deviation in a region devoid of spectral features, as shown in eq S1. This was done for both the corrected and uncorrected spectra. The RSD for the water vapor absorption region 1200 to 2100 cm*^–^*^1^ was calculated by dividing by the mean standard deviation in the 4800 to 5000 cm*^–^*^1^ which contains no spectral features. Likewise, the RSD in the CO_2_ absorption region 2300 to 2400 cm*^–^*^1^ was calculated by dividing by the mean standard deviation in the featureless 4800 to 5000 cm*^–^*^1^ region shown in Figure 4d. As can be seen in Figure 5, the standard deviation of the uncorrected spectra for both the water vapor (Figure 5a) and CO_2_ (Figure 5b) regions greatly exceeds that of the corrected spectra, showing the correction algorithm does effectively reduce standard deviation in regions of atmospheric absorption. The RSD of the corrected spectra is close to a value of one, which would be expected for a spectrum with no interferent absorptions.

### Spectral Resolution

**Figure 6:**
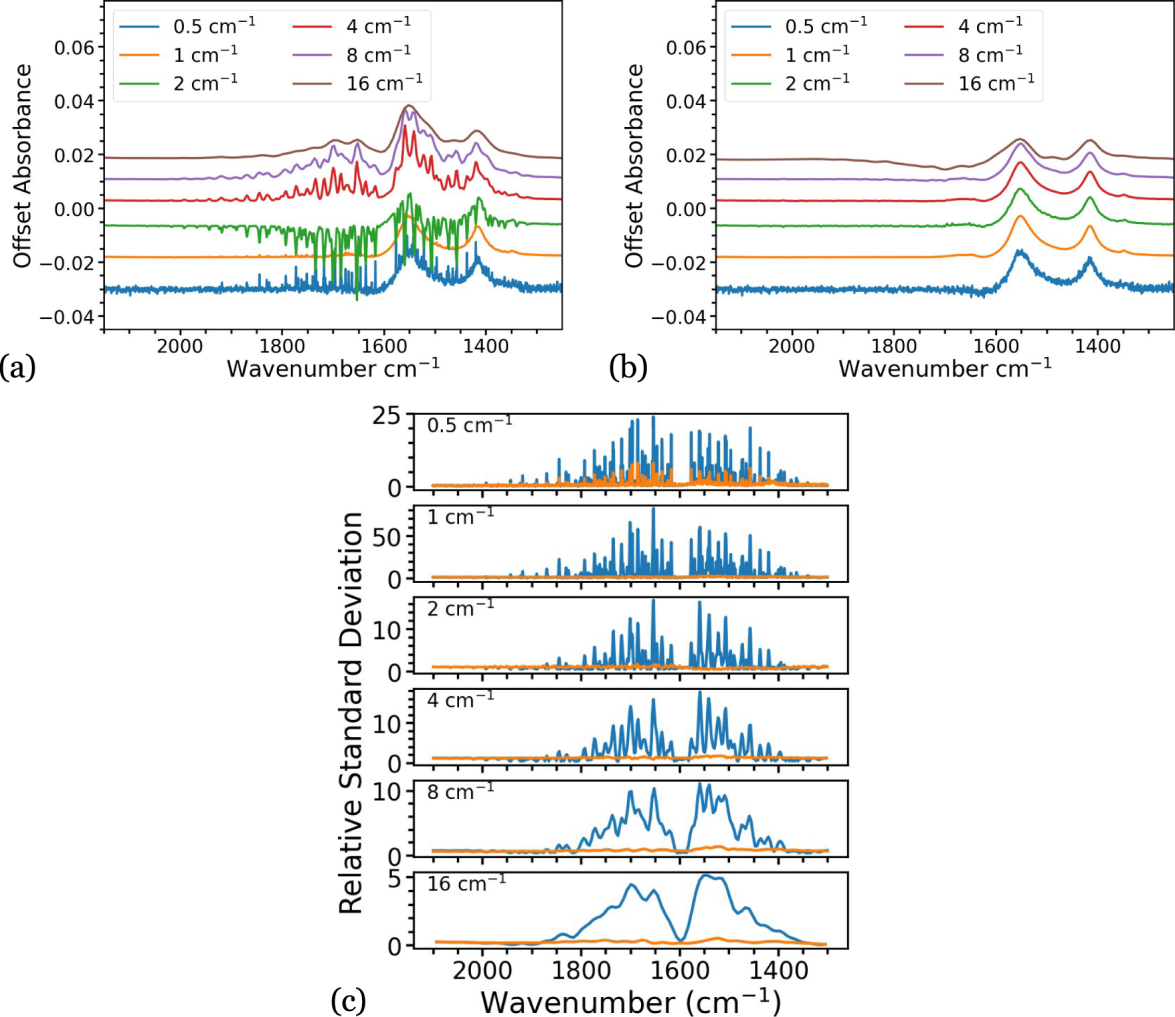
(a) Example spectra of sodium acetate at a range of resolutions showing water vapor interference. (b) The spectra in (a) are corrected for water vapor absorptions using the algorithm. (c) The RSD of the corrected (orange) and uncorrected (blue) spectra are shown for all resolutions.

The effect of spectral resolution on the efficacy of the algorithm was investigated by measuring the spectra of 250 mM sodium acetate (background corrected to water) at resolutions of 0.5 cm*^–^*^1^, 1 cm*^–^*^1^, 2 cm*^–^*^1^, 4 cm*^–^*^1^, 8 cm*^–^*^1^, and 16 cm*^–^*^1^. Nine spectra were measured at each resolution. Figure 6a shows an example uncorrected spectrum for each resolution, corrected using the subsequent spectrum (shown in Figure 6b). All example spectra show improvement upon correction, although the spectrum with 16 cm*^–^*^1^ resolution shows distortion both before and after correction, attributed to the fine structure of the water vapor not being properly resolved. To quantify the effectiveness of the method at each resolution, the RSD was calculated for each set of spectra at each respective resolution, using the methodology explained previously, with mean standard deviation in the feature-free 4800–5000 cm*^–^*^1^ region. These data are shown in Figure 6c. All spectra display a clear reduction in RSD following correction. However, the spectra collected at 0.5 cm*^–^*^1^ show the RSD greatly exceeding an ideal value around 1 following correction, indicating that this may be a limit for the application of this method. This is likely due to an instrumental drift over the time period of the scans, which is much longer than for lower resolution scans, resulting in a poorly defined difference spectrum and consequently a poorly corrected spectrum.

Weis and Ewing^11^ and Zhang et al.^5^ identified an additional problem associated with fluctuating temperature and pressure during measurements. They discussed that any method based on spectral subtraction is prone to error due to instability of the reference laser, due to fluctuations in environmental conditions. This effect can be exacerbated when second derivative spectra are used, as is commonly done in the development of machine learning models.^12^ Our use of two sequential spectra for correction has the added advantage of measurement over a short time period. This means that, assuming a relatively stable temperature environment, instability in the laser cavity due to changes in the temperature should be insignificant. Indeed, we have not observed this to be an issue with our technique as shown by the RSD calculations in Figure 5 for a resolution of 4 cm*^–^*^1^. However, this could be an additional reason for the reduced correction ability of the algorithm at the higher resolution of 0.5 cm*^–^*^1^ shown in Figure 6c, where measurements occur over a longer time frame.

### Machine Learning

To demonstrate that our method does provide a sufficient correction for usage in “real-world” scenarios using second derivative spectra, we apply machine learning to determine the DPPC—also known as lecithin—concentration in lecithin–sphingomyelin mixtures from spectra. The lecithin–sphingomyelin ratio is an important measurement for determining lung maturity in preterm infants, with a ratio above 2.2 indicating developed lungs.^13,14^ Binary mixtures of DPPC (concentration between 0 and 1.7 mM) and SM (concentration between 0 and 0.83 mM) were prepared to produce lecithin–sphingomyelin ratios between 0.0 and 8.3. A total of 824 IR absorption spectra were collected from these mixtures, background corrected to pure DCM, with each measurement run three times and each containing between 8 and 18 spectra. 80% of spectra were used as a training set, with the remainder set aside for testing after the model was established. All spectra in a single run were assigned to the same set such that the training and test sets could be independently corrected. For ease of automated calculation, each run lost one spectrum in the corrected spectra set, so the same spectra were removed from the uncorrected group to ensure that the models were trained on the same number of spectra. In total, there were 577 spectra in the training set and 166 spectra in the test set for both the corrected and uncorrected groups. The training set was further split into multiple cross validation training and test sets. Second derivative spectra from these groups were then used to train a partial least-squares regression (PLSR) model to predict lecithin concentration in each lecithin-sphingomyelin mixture. Example corrected and uncorrected spectra (original and second derivative) for a lecithin–sphingomyelin mixture are shown in Figures S3 and S4. A model was selected for each corrected and uncorrected group containing the optimal number of latent variables (LVs); this was found by determining whether adding a latent variable to the model significantly improved the model performance. To do this, the prediction error sum of squares (PRESS) for the less complex model was compared to the model that provided the minimum PRESS^15^ in the cross-validation test set, and the significance assessed using the F-statistic.

**Figure 7:**
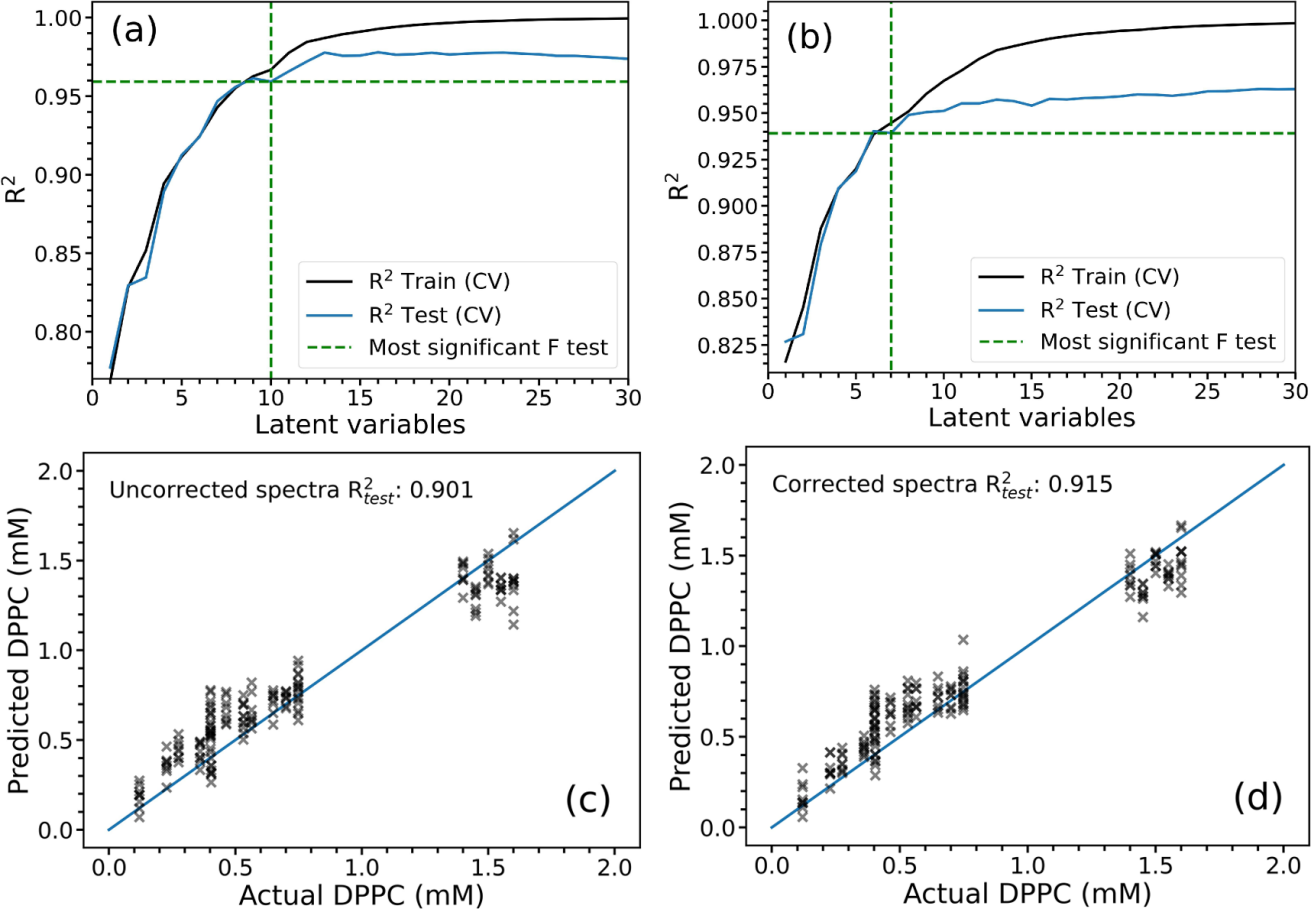
(a,b) Cross validation averaged *R*^2^ outputs used to establish the optimal number of LVs for each PLSR model. (a) shows the performance of the uncorrected spectra, while (b) shows the corrected spectra. A fewer number of LVs were required by the PLSR model of the corrected spectra (7 LVs) than the uncorrected spectra (10 LVs) based on the F-test ratio method for selecting the optimal number of LVs. Graphs below show tests for PLSR models of the (c) uncorrected and (d) corrected spectra showing similar performance for both models.

It was found that the PLSR model trained on the uncorrected second derivative spectra was best described by a model containing 10 LVs (Figure 7a) (*p* = 0.9), whereas only 7 LVs were required for the model for the corrected second derivative spectra (Figure 7b). These models were then retrained on the entire training data set and used to predict the concentration of lecithin in the test spectra. Figure 7c,d shows the results of implementing the PLSR model on the test set, with coefficient of determination slightly higher for corrected  than  uncorrected spectra. However, this small difference is unlikely to be significant. Of more importance is the 3 fewer LVs required to best describe the corrected second derivative spectra than the uncorrected second derivative spectra. This means that the effect of the correction is to permit the specification of a more parsimonious model, which is less prone to overfitting. Thus, this correction algorithm is effective when it is used on spectra that are used as second derivatives to produce machine learning models.

## Summary

Water vapor and carbon dioxide absorptions in the mid-infrared pose a challenge for FTIR spectroscopic measurements. Changing concentrations of these absorbants during the time frame of a background and sample measurement result in residual interference in spectra, including in regions important for fingerprint measurements of biomolecules. Several methods have previously been produced to correct for this interference; however, many place the burden of additional measurements on the researcher or require careful control of environmental conditions not possible outside of a laboratory setting. We presented a method that overcomes these issues by only requiring two sequentially measured sample spectra. The difference of these spectra, multiplied by a scaling factor optimized by minimizing point-to-point length of the spectrum optimized using a loss function, is used to correct regions of atmospheric absorption. Its effectiveness in removing interference from water vapor and CO_2_ has been proven in correcting the spectra of sphingomyelin and benzaldehyde, which includes sharp intense peaks that challenged the approach. Furthermore, corrected second derivative spectra were shown to produce more parsimonious PLSR models, which are less prone to overfitting. This opens the door for analysis of spectral regions that can be difficult to interpret due to atmospheric interference unless measurements are made under carefully controlled conditions. Furthermore, the simple implementation of this algorithm lends itself to automation in medical point-of-care or nonlaboratory based measurements such as industrial process monitoring by nonexpert end users.

## Supporting Information Available

Diagram demonstrating calculation of point-to-point spectral length, additional examples of corrected spectra with minor water vapor interference, explanations of calculations of relative standard deviation, and example spectra used for machine learning (PDF)
