# Supplementary material for: A Rapid and Simplified Approach to Correct Atmospheric Absorptions in Infrared Spectra
Source: Anal Chem. 2024 Oct 31;96(45):18052–60. doi: 10.1021/acs.analchem.4c03594 (PMC11561874; doi:10.1021/acs.analchem.4c03594)
Supplement: Supplementary file 1 — ac4c03594_si_001.pdf [file ac4c03594_si_001.pdf]

Supporting information

A Rapid and Simplified Approach to Correct Atmospheric Absorptions in Infrared Spectra

Waseem Ahmed,[†,‡] Eleanor L. Osborne,[†,‡] Aneesh Vincent Veluthandath,[†] and

Ganapathy Senthil Murugan[,†]*

[†]Optoelectronics Research Centre, University of Southampton, Southampton, SO17 1BJ, United Kingdom

[‡]These authors contributed equally.

Table of Contents

A. Point-to-point length S-2
B. Correcting small differences S-2, S-3
C. Relative standard deviation calculation S-3
D. L/S spectrum used for machine learning S-3, S-4

A. Point-to-point length

The calculation of point-to-point length is demonstrated in Figure S1.

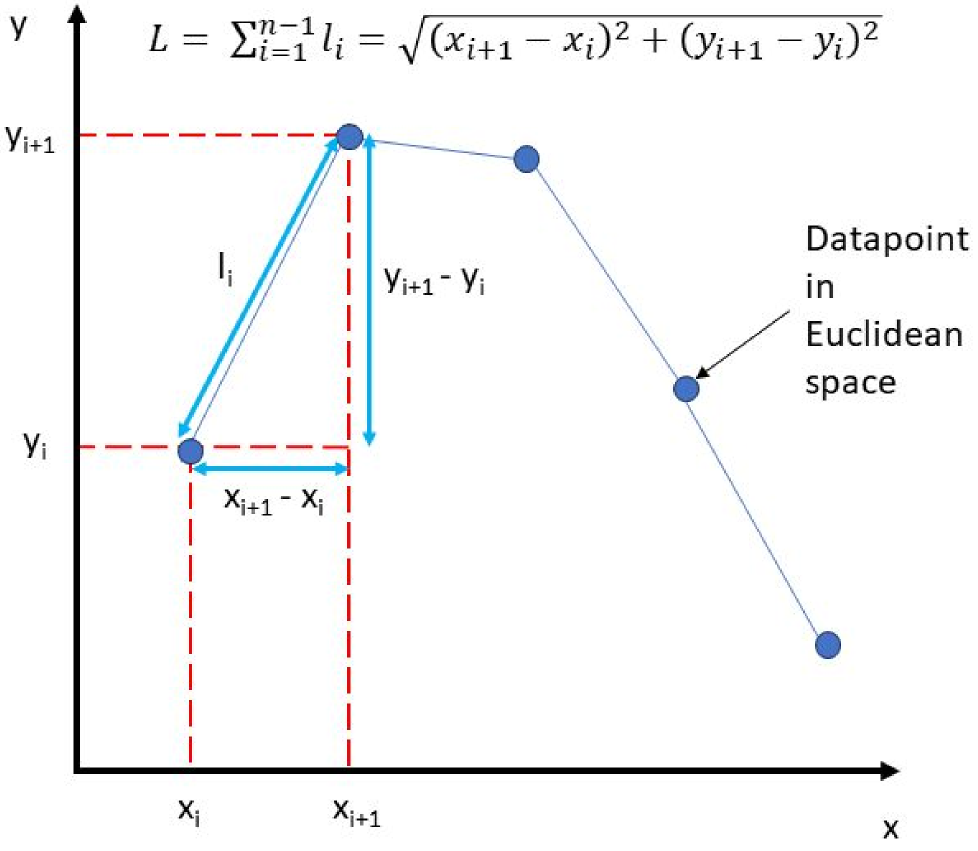

Figure S1: Diagram showing point-to-point distance calculation. Each datapoint is mapped to Euclidean space with unitless coordinates (x,y). Pythagorean theorem is used to calculate the distance between consecutive datapoints.

B. Correcting small differences

In all cases, there will be a scaling factor that gives a minimum point-to-point spectral length for the scaled subtraction of the difference spectrum from the uncorrected spectrum, as shown in Figure 2c. This was additionally tested with benzaldehyde ideal spectra for which the point-to-point spectral length of the uncorrected spectrum in the water vapour correction region was calculated as 769.7576095514835. The corrected spectrum was calculated using the subsequent spectrum (where the variation in water is subtle), and its length was calculated to be 769.7576183648075, which indicates that small corrections were in fact made, as shown in Figure S2. If the same spectrum is used to correct itself instead of the subsequent spectrum, the search for the optimised gamma immediately terminates, because the difference spectrum is zero.

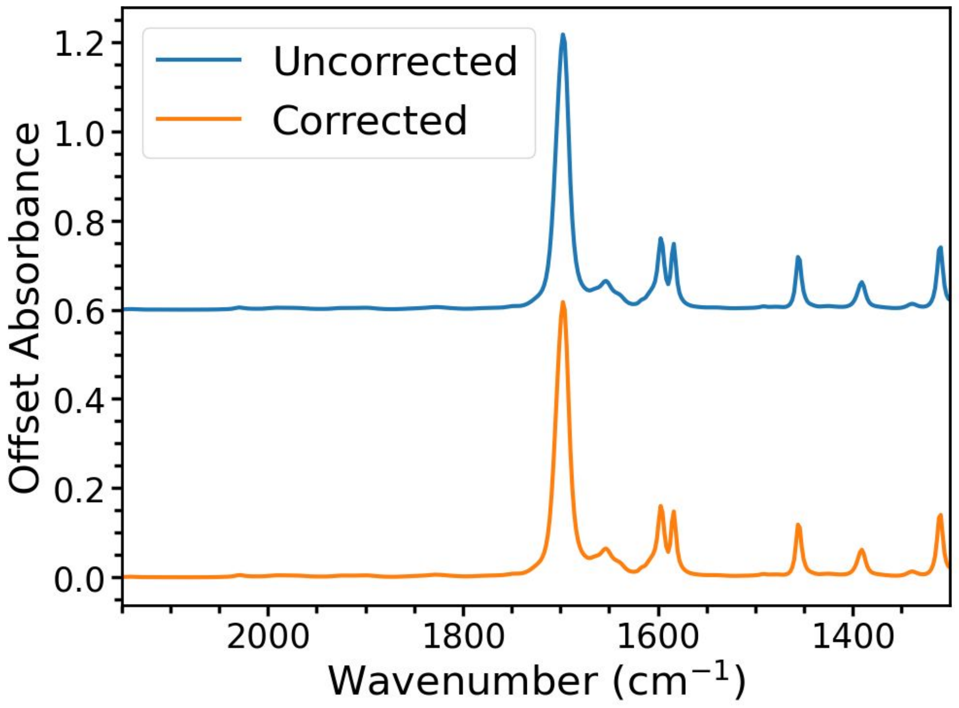

Figure S2: An uncorrected benzaldehyde spectrum shown with negligible water vapour absorbances, allowing its classification as an 'ideal' spectrum, is corrected for water vapour using the algorithm. The corrected spectrum retains analyte features without any interferent features showing.

C. Relative standard deviation calculation

$$RSD_i = \frac{\sigma_i}{\sum_{k=1}^{m}\frac{\sigma_k}{m}} = \frac{\sqrt{\sum_{j=1}^{n}(y_{i,j}-\mu_i)^2}}{\sum_{k=1}^{m}\frac{1}{m}\sqrt{\sum_{j=1}^{n}(y_{k,j}-\mu_k)^2}} \quad (1)$$

σ_i is the standard deviation at each index i in the corrected region, σ_k is the standard deviation calculated across the featureless baseline region at each index k, j is the index of the spectrum in the total n corrected spectra, $y_{i,j}$ is the value of absorbance at data point i in the corrected region, in spectrum j, $\mu_i$is the mean value of absorbance at each data point i, m is the number of datapoints in the baseline region of a spectrum, $y_{k,j}$ is the value of absorbance at datapoint k in spectrum j, and μ_k is the mean absorbance in the baseline region at each datapoint k.

D. L/S spectrum used for machine learning

An example spectrum from machine learning section is shown in Figure S3:

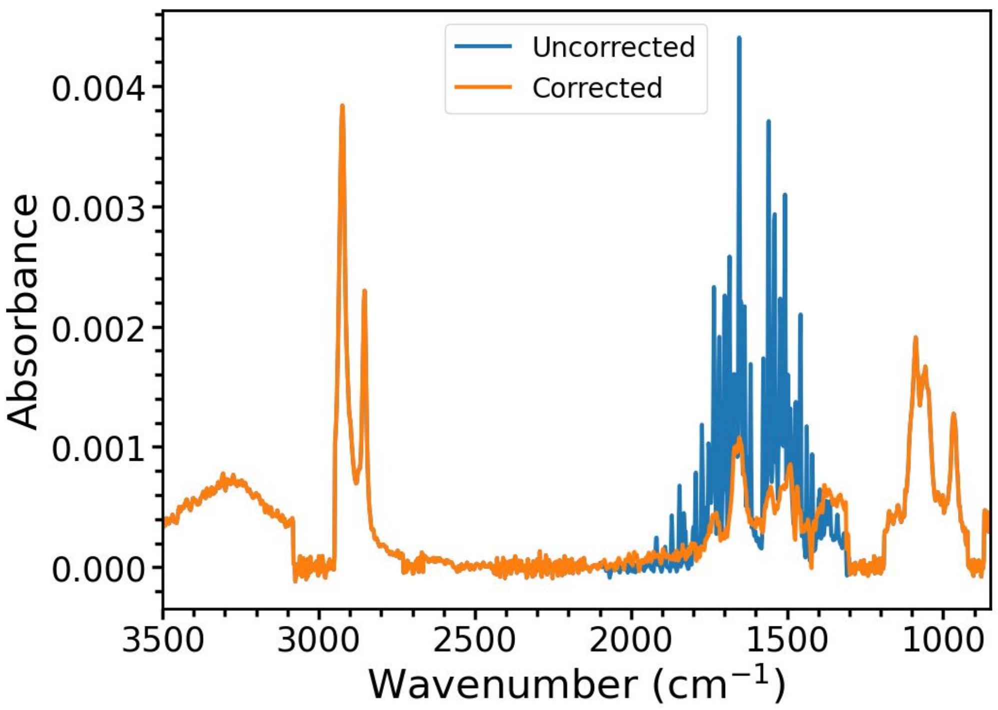

Figure S3: Uncorrected and corrected spectra for an analyte mixture of DPPC concentration of 0.22758 mM and sphingomyelin concentration of 0.549501 mM used to train the PLSR models.

For the purpose of generating the PLSR models, the second derivative dataset was used, and the spectra above were observed to be as per the spectra in Figure S4:

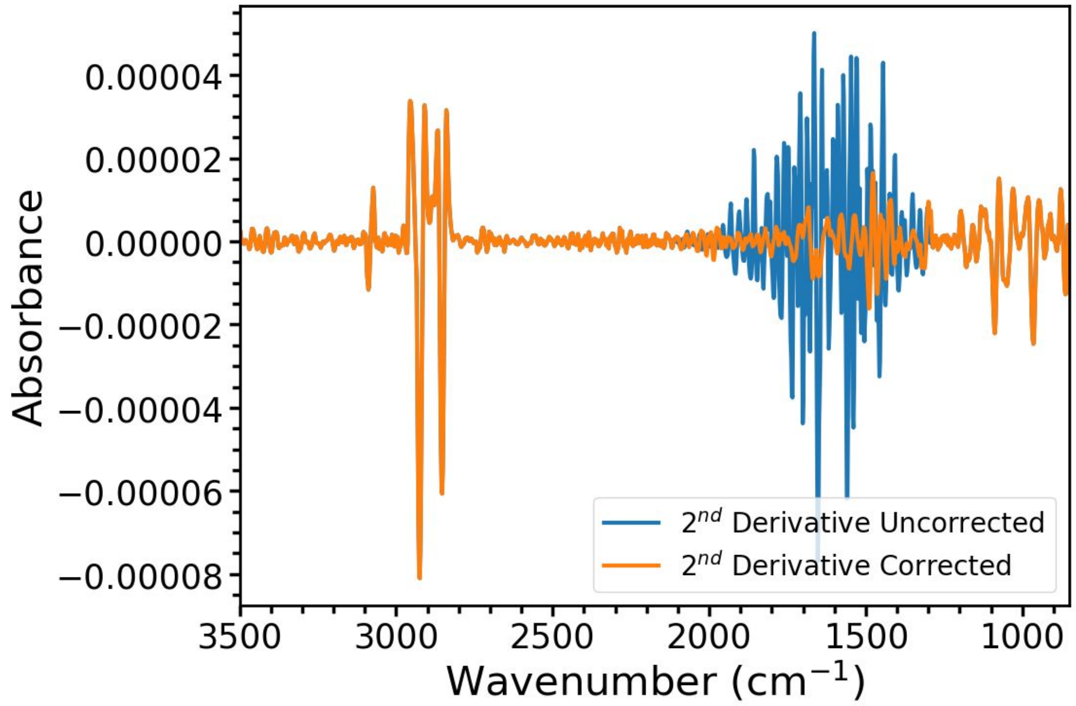

Figure S4: Uncorrected and corrected second derivative spectra for an analyte mixture of DPPC concentration of 0.22758 mM and sphingomyelin concentration of 0.549501 mM used to train the PLSR models.
